# Cutting Modeling of Hybrid CFRP/Ti Composite with Induced Damage Analysis

**DOI:** 10.3390/ma9010022

**Published:** 2016-01-04

**Authors:** Jinyang Xu, Mohamed El Mansori

**Affiliations:** MSMP—EA 7350 Laboratoire, Arts et Métiers ParisTech, Rue Saint Dominique B.P. 508, 51006 Châlons-en-Champagne, France; mohamed.elmansori@ensam.eu

**Keywords:** hybrid composite, FE modeling, orthogonal cutting, induced damage, interface delamination, fiber/matrix failure

## Abstract

In hybrid carbon fiber reinforced polymer (CFRP)/Ti machining, the bi-material interface is the weakest region vulnerable to severe damage formation when the tool cutting from one phase to another phase and *vice versa*. The interface delamination as well as the composite-phase damage is the most serious failure dominating the bi-material machining. In this paper, an original finite element (FE) model was developed to inspect the key mechanisms governing the induced damage formation when cutting this multi-phase material. The hybrid composite model was constructed by establishing three disparate physical constituents, *i.e.*, the Ti phase, the interface, and the CFRP phase. Different constitutive laws and damage criteria were implemented to build up the entire cutting behavior of the bi-material system. The developed orthogonal cutting (OC) model aims to characterize the dynamic mechanisms of interface delamination formation and the affected interface zone (AIZ). Special focus was made on the quantitative analyses of the parametric effects on the interface delamination and composite-phase damage. The numerical results highlighted the pivotal role of AIZ in affecting the formation of interface delamination, and the significant impacts of feed rate and cutting speed on delamination extent and fiber/matrix failure.

## 1. Introduction

Hybrid composites, especially those carbon fiber reinforced polymer (CFRP)/Ti stacks, have been identified as an innovative structural configuration in the modern aerospace industry. The enhanced mechanical properties and improved structural functions have given the material a high demand for manufacturing key aircraft structures subjected to high thermo-mechanical stresses. A typical application is the use of wing-fuselage connections in the new-generation Boeing 787 Dreamliner. The CFRP-to-Ti coupling typically provides the best combination of metallurgical and physical properties including high strength-to-weight ratio, low density, and superior corrosion/erosion resistance that favor energy saving in industrial applications [1,2,3]. Generally, the CFRP/Ti composite exhibits a high strength-to-weight ratio with yield strength as high as 830 MPa and a density of roughly 4 g/cm^3^ [4].

Prior to their post applications, structural components made of hybrid CFRP/Ti composite are mostly manufactured in near-net-shape in order to achieve dimensional tolerance and assembly requirement. However, due to the disparate machinability behaviors of each stacked constituent, the manufacturing hybrid composite exhibits the most challenging task in industrial sectors. For instance, the titanium phase exhibits high mechanical properties, low thermal conductivity, and strong chemical affinity to tool materials, which commonly results in high force/heat generation, serious tool wear (abrasive wear and adhesion wear), and short tool life [5,6,7]. In contrast, the CFRP phase shows anisotropic behavior, abrasive nature, and low thermal conductivity, which leads to severe subsurface damage, poor heat dissipation, and excessive tool wear [8,9,10].

In hybrid CFRP/Ti machining, typically three cutting stages are involved, *i.e*., the Ti-phase cutting, interface cutting, and CFRP-phase cutting. Among them, the interface region (also refer to the “Ti-to-CFRP” contact boundary) represents the most difficult-to-cut zone vulnerable to severe damage formation when the tool edges are cutting from one phase (Ti phase) to another phase (CFRP phase) and *vice versa*. The interface region is usually characterized as a physically intermediate transition zone that really exists in the bi-material machining process. During interface cutting, the interface area usually suffers changeable chip-separation modes and experiences severe mechanical/physical phenomena transition exerted at the bi-material contact boundary. In such circumstances, the interface region becomes the most challenging cutting zone as compared to absolute Ti-phase cutting and absolute CFRP-phase cutting while machining the hybrid composite. The discontinuity of the tool-work interaction governing interface cutting commonly makes the machining behavior more complicated and interrelated. Inspections of interface damage in CFRP/Ti cutting via the experimental method have been shown to be very challenging and highly difficult [11]. Despite the fact that several experimental investigations [1,2,12] have been well performed, some key issues have still not been clearly addressed: (*i*) the key mechanisms and physical phenomena controlling the CFRP/Ti interface cutting, (*ii*) the parametric effects on the interface machining and subsequently induced damage extent, and (*iii*) the machinability classification of hybrid CFRP/Ti machining, *i.e*., which region of cutting actually reflects the machinability of pure Ti-phase cutting (*M*_Ti_), stacked material cutting (*M*_CFRP/Ti_), and pure CFRP-phase cutting (*M*_CFRP_), respectively. In addition, the conventional experimental method is cost-prohibitive and time-consuming. In contrast, the numerical approach should be a qualified tool helpful to enable feasible inspections of the damage formation when cutting this bi-material. Furthermore, although a considerable amount of scientific work has dealt with single Ti-cutting modeling and single CFRP-cutting modeling, comprehensive numerical studies concerning hybrid CFRP/Ti machining have still been only rarely reported.

These are the key incentives that motivated the current work to develop an original finite element (FE) model to address the mentioned topics. To inspect the fundamental mechanisms controlling the bi-material machining, the simplified orthogonal cutting configuration (OCC) was adopted. The OCC represents a convenient way to reveal the most fundamental machining physics governing the various actual manufacturing operations of hybrid CFRP/Ti composite, e.g., drilling, grinding, *etc*. The key objective of this investigation aims to establish an FE model for damage predictions and failure analyses when orthogonally cutting hybrid CFRP/Ti composite. The established numerical model incorporated three physical constituents, *i.e*., the Ti phase, CFRP phase, and interface layer. The CFRP/Ti model was rigorously validated prior to its post-application. The multiple aspects of machining responses including cutting process, interface delamination, and subsurface damage formation were precisely investigated via finite element (FE) analyses. A particular concentration was made to characterize the dynamic process of delamination formation and affected interface zone (AIZ). The numerical results highlighted the significant role of AIZ and bi-material interface consumption (BIC) in controlling the induced interface damage formation.

## 2. Orthogonal Cutting (OC) Model for Hybrid CFRP/Ti Cutting

### 2.1. Numerical Setup of the OC Model

**Figure 1:**
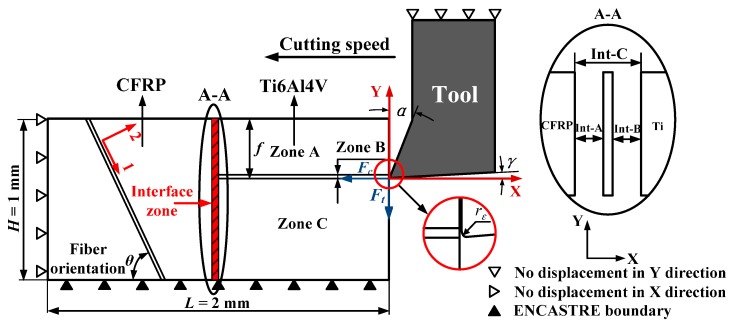
Scheme of the established orthogonal cutting (OC) model for hybrid CFRP/Ti machining (α = 12° and γ = 7°): (1,2) represents the material coordinate system where 1 → fiber direction, 2 → transverse direction, *F_c_* indicates the cutting force and *F_t_* signifies the thrust force (ENCASTRE boundary indicates the fully fixed end). (see Abbreviations Section).

In the current work, a 2D orthogonal cutting model was developed by using the commercial software Abaqus/Explicit code (Version 6.11, Dassault Simulia, Paris, France). To simulate the hybrid cutting operation, the machining process was specified as shown schematically in Figure 1. The basic geometries of the tool-workpiece couple and boundary condition are illustrated in Figure 1. The established orthogonal cutting (OC) model is comprised of four basic phases, *i.e*., the tool part, Ti part, interface part, and CFRP part with total dimensions of 2 mm × 1 mm (*L × H*) for the workpiece material.

The cutting tool was modeled as a rigid body and imposed by a cutting velocity on its reference node toward the horizontal direction (*i.e*., negative *X*-axis) to finalize the cutting simulation. The tool was configured by defined geometries of rake and clearance angles (α = 12° and γ *=* 7°) as depicted in Figure 1. The center of the tool tip was placed exactly at the feed-rate distance from the upper surface. Fixed displacements were applied on both the bottom and left edges of the stack model. The bottom edge of the OC model was restrained in all directions (ENCASTRE) while the left edge was constrained to move along the horizontal direction (*X* direction), as shown in Figure 1.

The Ti phase was modeled as a fully isotropic and homogeneous material. A four-node plane-strain thermally coupled quadrilateral element type CPE4RT, which has better convergence properties was utilized for a coupled temperature-displacement analysis and enhanced hourglass control was selected for the whole set of the Ti elements. The entire Ti phase was divided into three physical zones: (*i*) Zone A denoted the separated chip layer, (*ii*) Zone B signified the predefined separation path (joint layer) and (*iii*) Zone C represented the machined Ti surface, where mesh generations exhibited different characteristics. Both Zones A and B were defined by very fine mesh density, whereas Zone C was constructed by coarse mesh element density far away from the vicinity of the tool-work contact region. The surface-to-surface contact algorithm was used to model the interaction between the cutting tool, Zone A and Zone B. The kinematic contact algorithm was assigned to the contact pairs in order to avoid element penetrations. Friction in the orthogonal cutting commonly occurs at the contact surfaces of tool and workpiece, rake face and chip surface. The frictional shearing stress is the average of the shearing stress at the tool and chip interface. In the present work, the Coulomb’s friction law was utilized to describe the contact behavior.

For the CFRP part, the composite in reality consists of two distinct phases (fiber and matrix) and globally exhibits anisotropic properties. However, for simulations in a macro-mechanical model, the reinforced laminate has commonly been assumed as an equivalent homogeneous material (EHM) by most numerical study cases [13,14,15,16]. In the present model, the CFRP layer was modeled as EHM by using four-node plane-stress linearly interpolated elements (CPS4R) with reduced integration and automatic hourglass control. It should be noted that a plane strain analysis, which is used typically for metal cutting, was not appropriate for machining CFRP laminates due to the extent of out of plane material displacement observed in the cutting experiments [17]. The interaction between the CFRP phase and tool was regulated by the algorithm surface-node-surface contact available in the Abaqus/Explicit code.

To link the Ti phase and CFRP phase together, an interface layer was introduced in the FE model and simulated as a quick transition zone by using a cohesive element. It should be stressed that the use of the interface layer here serves as a technical control for the “Ti-to-CFRP” contact management during simulation. A triangular traction-separation cohesive formulation with linear softening was used to represent its mechanical response. The assembly of the interface layer with both the Ti phase and CFRP phase was carried out by setting a constraint type join (tie constraint). Furthermore, two contact pairs (Int-A and Int-B) with specifications of penalty contact algorithm and rough friction formulation were established in the interface zone, Ti phase and CFRP phase as shown in Figure 1, which made the interface zone a slave surface. Moreover, an additional contact pair referring to the Int-C (as shown in Figure 1) was also assigned between the Ti phase and CFRP phase in order to avoid them penetrating each other when the cohesive elements were eroded.

### 2.2. Ti-phase Model

The material behavior of the Ti phase was assumed to be isotropic and elastic-plastic with thermal softening by using the isotropic plasticity model available in the Abaqus/Explicit code. The material properties for the Ti phase (Ti6Al4V) are summarized in Table 1 [18]. The Young’s modulus (*E*), thermal expansion coefficient (α*_T_*), thermal conductivity (λ) and specific heat (*c_p_*) were considered to be temperature-dependent in order to accurately represent the property variation of titanium phase *versus* thermal influence during the cutting process.

**Table 1:** Mechanical properties of the Ti6Al4V phase [18].

Physical Parameter	Ti6Al4V
Density (ρ)	4430 (kg/m^3^)
Young’s modulus (*E*)	E=0.7412T+113.375 (GPa)
Poisson’s ratio (*υ*)	0.342
Thermal expansion coefficient (α*_T_*)	$\alpha_{T}=2\times 10^{-9}\times T+9\times 10^{-6}$ (°C^−1^)
Melting temperature (*T_m_*)	1680 (°C)
Room temperature (*T_r_*)	25 (°C)
Thermal conductivity (λ)	$\lambda =7.039e^{0.0011T}$ (W/(m·°C))
Specific heat (*c_p_*)	$c_{p}=2.24e^{0.0007T}\times 10^{6}/\rho$ (J/(kg·°C))

Note*:* the term “*T*” indicates the cutting temperature generated inside the Ti6Al4V alloy during the machining process.

In FE modeling, accurate material flow stress models are very much required to capture the constitutive behavior of the work material under high strain/strain rate/temperature encountered in machining. The constitutive model proposed by Johnson-Cook (JC) [19,20] was applied in this investigation, which offers a satisfactory description of ductile material behavior by considering large strains, high strain rates, and temperature-dependent visco-plasticity encountered in machining. The JC material model also takes into account the effects of strain hardening, strain rate sensitivity, and thermal softening behavior as illustrated in Equation (1).

(1) $$\bar{\sigma }=\underset{Strain\;hardening}{\underset{︸}{\left(A+B{\bar{\varepsilon }}^{n}\right)}}\underset{Strain\text{​ }rate\;sensitivity}{\underset{︸}{\left(1+C\ln\frac{\dot{\bar{\varepsilon }}}{{\dot{\bar{\varepsilon }}}_{0}}\right)}}\underset{Thermal\;softening\;behavior}{\underset{︸}{\left[1-{\left(\frac{T-T_{r}}{T_{m}-T_{r}}\right)}^{m}\right]}}$$

Where $\bar{\sigma }$ denotes the equivalent flow stress, $\bar{\varepsilon }$ is the equivalent plastic strain, $\dot{\bar{\varepsilon }}$ is the equivalent plastic strain rate, ${\dot{\bar{\varepsilon }}}_{0}$ is the reference equivalent plastic strain rate, *T* is the cutting temperature of Ti6Al4V alloy during the machining process, *T_m_* is the material melting temperature, and *T_r_* is the room temperature. *A*, *B*, *C*, *m* and *n* are material constants, which are usually determined by fitting the strain-stress curves obtained by split-Hopkinson bar. In this simulation, the JC material constants are selected carefully from the open literature [21,22] as shown in Table 2.

**Figure 2:**
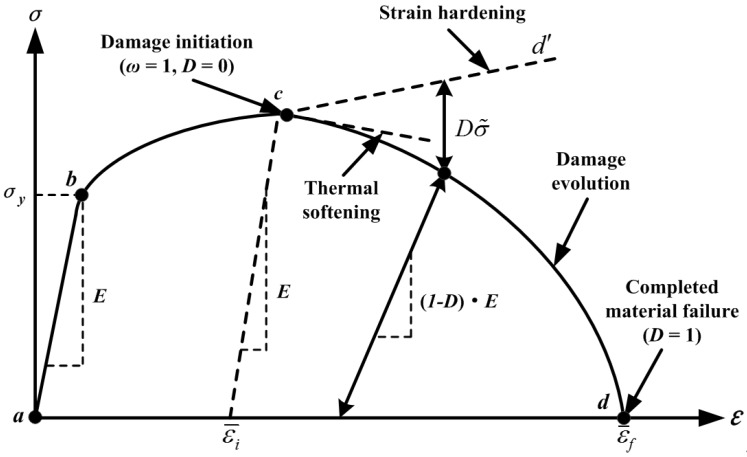
Typical uniaxial strain–stress (ε–σ) responses of ductile material failure process [23]. (Note: $\sigma_{y}$ is the yield stress, $\tilde{\sigma }$ is the effective (or undamaged) flow stress, ${\bar{\varepsilon }}_{i}$ implies the equivalent plastic strain at damage initiation and ${\bar{\varepsilon }}_{f}$ indicates the equivalent plastic strain at failure).

In order to simulate the chip separation process, an energy-based ductile failure criterion was applied in the FE computation. The failure damage criteria consist of two-stage laws, *i.e*., failure initiation law and failure evolution law, to describe the failure responses of ductile material as shown in Figure 2 [23]. The failure formation includes two steps described below.

**Table 2:** Input parameters for Johnson-Cook (JC) constitutive model and JC damage law [21,22].

JC Model Type	JC Model Parameter				
JC constitutive model	*A* (MPa)	*B* (MPa)	C	*n*	*m*
	1098	1092	0.014	0.93	1.1
JC damage law	*D_1_*	*D_2_*	*D_3_*	*D_4_*	*D_5_*
	−0.09	0.25	−0.5	0.014	3.87

*Step 1: Damage Initiation.* The JC failure model was used as a damage initiation criterion, which contains five failure parameters that need to be determined (*D*_1_–*D*_5_) as presented in Equation (2). In the JC failure model, damage initiation is assumed to happen when a scalar damage parameter (*ω*) reaches 1. The *ω* parameter is defined based on a cumulative law as described in Equation (3).

(2) $${\bar{\varepsilon }}_{i}=\left[D_{1}+D_{2}\exp\left(D_{3}\frac{P}{\bar{\sigma }}\right)\right]\left(1+D_{4}\ln\frac{\dot{\bar{\varepsilon }}}{{\dot{\bar{\varepsilon }}}_{0}}\right)\left[1+D_{5}\left(\frac{T-T_{r}}{T_{m}-T_{r}}\right)\right]$$

(3) $$\omega =\sum \frac{\Delta \bar{\varepsilon }}{{\bar{\varepsilon }}_{i}}$$

In the above equations, ${\bar{\varepsilon }}_{i}$ is the equivalent plastic strain at damage initiation, *P* is the hydrostatic pressure, $P/\bar{\sigma }$ is the stress triaxiality, *D_1_*–*D_5_* are JC damage parameters, *ω* is the scalar damage parameter and $\Delta \bar{\varepsilon }$ is the equivalent plastic strain increment. The following parameters summarized in Table 2 [21] were adopted for *D_1_*–*D_5_* in the cutting simulation.

*Step 2: Damage Evolution.* When the ductile material damage is initiated, the strain-stress relationship no longer accurately represents the real material behavior. Based on this, Hillerborg’s fracture energy proposal [24] was used to reduce mesh dependency by creating a displacement-stress response after damage initiation. Hillerborg defines the energy required to open a unit area of crack (*G_f_*) as a material parameter, and the fracture energy is represented as follows:

(4) $$G_{f}=\int_{{\bar{\varepsilon }}_{i}}^{{\bar{\varepsilon }}_{f}}L\sigma_{y}d\bar{\varepsilon }=\int_{0}^{{\bar{u}}_{f}}\sigma_{y}d\bar{u}$$

In the equation, ${\bar{\varepsilon }}_{i}$ is the equivalent plastic strain at damage initiation, ${\bar{\varepsilon }}_{f}$ is the equivalent plastic strain at failure, *L* denotes the characteristic length, $\sigma_{y}$ signifies the yield stress, $\bar{\varepsilon }$ is the equivalent plastic strain, ${\bar{u}}_{f}$ is the equivalent plastic displacement at failure, and $\bar{u}$ is the equivalent plastic displacement.

The FE model applied the planar quadrilateral continuum element (CPE4RT), and then characteristic length (*L*) was defined as a half typical length of a line across a second order element. As the direction in which fracture occurs was not known in advance, so the definition of characteristic length was used.

A linear damage parameter (*D_l_*) was used for the joint layer according to the following equation:

(5) $$D_{l}=\frac{L\bar{\varepsilon }}{{\bar{u}}_{f}}=\frac{\bar{u}}{{\bar{u}}_{f}}$$

Where the equivalent plastic displacement at failure (${\bar{u}}_{f}$) was computed as follows:

(6) $${\bar{u}}_{f}=\frac{2G_{f}}{\sigma_{y}}$$

In contrast, an exponential damage parameter (*D_e_*) was used for the chip layer according to the following equation:

(7) $$D_{e}=1-\exp\left(-\int_{0}^{\bar{u}}\frac{\bar{\sigma }}{G_{f}}d\bar{u}\right)$$

At any given time during the FE calculation, the equivalent flow stress in the material is given by the following equation:

(8) $$\bar{\sigma }=\left(1-D\right)\tilde{\sigma }$$

Where $\tilde{\sigma }$ denotes the effective (or undamaged) equivalent flow stress computed in the current increment and *D* represents the damage parameter (*D_l_* or *D_e_*).

In this study, *G_f_* is provided as an input parameter and theoretically is a function of Poisson’s ratio (*υ*), Young’s modulus (*E*), and fracture toughness (*K_C_*) as shown in Equation (9). Considering the different fracture mechanics [25] occurring on the chip-separation process, two different values of fracture energy were utilized as input data in the Abaqus/Explicit code: (*G_f_*)*_I_* for the joint layer (Zone B) and (*G_f_*)*_II_* for the chip layer (Zone A). The (*G_f_*)*_I_* denotes the fracture energy of mode I which is a tensile mode (opening mode normal to the plane of the fracture) whereas (*G_f_*)*_II_* signifies the fracture energy of mode II which is a shearing one (sliding mode acting parallel to the plane of the fracture).

(9) $${\left(G_{f}\right)}_{I,II}=\left(\frac{1-\nu^{2}}{E}\right){\left(K_{C}^{2}\right)}_{I,II}$$

### 2.3. CFRP-Phase Model

For CFRP phase, the simulated material was unidirectional carbon/epoxy T300/914 laminate (T300/914 represents the standard specification of the used CFRP material) and its mechanical/physical properties are summarized in Table 3 [26,27,28]. The definition of composite fiber orientation (θ) was made based on the introduction of the material coordinate system into the CFRP phase as shown schematically in Figure 1. To replicate the rupture of the fiber/matrix system, Hashin damage criteria [29] were adopted for the numerical computation. The Hashin damage criteria take into account four fundamental failure modes commonly occurring in composite machining, *i.e.,* fiber-tensile failure, fiber-compression failure, matrix-tensile failure, and matrix-compression failure, as summarized in Table 4. In Table 4, σ_11_ signifies the stress in the fiber direction, σ_22_ denotes the stress in the transverse direction, and σ_12_ represents the in-plane shear stress.

**Table 3:** Material properties of T300/914 CFRP used in simulation [26,27,28].

Material Properties	CFRP
Longitudinal modulus, *E*_1_ (GPa)	136.6
Transverse modulus, *E*_2_ (GPa)	9.6
In-plane shear modulus, *G*_12_ (GPa)	5.2
Major Poisson’s ratio, *υ*_12_	0.29
Longitudinal tensile strength, *X_T_* (MPa)	1500
Longitudinal compressive strength, *X_C_* (MPa)	900
Transverse tensile strength, *Y_T _* (MPa)	27
Transverse compressive strength, *Y_C_* (MPa)	200
In-plane shear strength, *S*_12_ (MPa)	80
Longitudinal shear strength, *S_L_* (MPa)	80
Transverse shear strength, *S_T_* (MPa)	60

The element erosion of the CFRP phase is conducted through the concept of stiffness degradation., *i.e*., when one type of the fiber/matrix failure occurs, the relative material properties will be degraded as shown in Table 4. In the present analysis, the material property degradation depends on four associated defined variables, as listed below: (*i*) the first variable noted HSNFTCRT represents the fiber-tensile failure mode; (*ii*) the second HSNFCCRT represents the fiber-compression failure mode; (*iii*) the third HSNMTCRT represents the matrix-tensile failure made and (*iv*) the fourth HSNMCCRT represents the matrix-compression failure mode.

**Table 4:** General formulation of 2D Hashin damage criteria for the CFRP phase [29].

Failure Criteria	Failure Mode	Associated Defined Variable	Reduced Material Properties
Fiber-tensile failure ($\sigma_{11}\geq 0$)	$D_{ft}^{2}={\left(\frac{\sigma_{11}}{X_{T}}\right)}^{2}+{\left(\frac{\sigma_{12}}{S_{L}}\right)}^{2}$	HSNFTCRT	*E*_1_, *E*_2_, *G*_12_, *υ*_12_ → 0
Fiber-compression failure ($\sigma_{11}<0$)	$D_{fc}^{2}={\left(\frac{\sigma_{11}}{X_{C}}\right)}^{2}$	HSNFCCRT	*E*_1_, *E*_2_, *G*_12_, *υ*_12_ → 0
Matrix-tensile failure ($\sigma_{22}\geq 0$)	$D_{mt}^{2}={\left(\frac{\sigma_{22}}{Y_{T}}\right)}^{2}+{\left(\frac{\sigma_{12}}{S_{L}}\right)}^{2}$	HSNMTCRT	*E*_2_, *G*_12_ → 0
Matrix-compression failure ($\sigma_{22}<0$)	$D_{mc}^{2}={\left(\frac{\sigma_{22}}{2S_{T}}\right)}^{2}+\left[{\left(\frac{Y_{C}}{2S_{T}}\right)}^{2}-1\right]\frac{\sigma_{22}}{Y_{C}}+{\left(\frac{\sigma_{12}}{S_{L}}\right)}^{2}$	HSNMCCRT	*E*_2_, *G*_12_ → 0

During the FE computation, the material properties at each integration point were evaluated and degraded depending on which set of failure mode was used. If any failure index reached unity, the relevant material properties were automatically reduced to zero according to the implemented stiffness degradation scheme. The procedure was repeated until the occurrence of complete chip formation.

### 2.4. Interface Model

The interface model used here aims to serve as a technical control for the “Ti-to-CFRP” contact management and to facilitate the characterization of interface damage formation during the simulation. It should be noted that in real CFRP/Ti configurations, some of them do not exist with such a third layer and only combine together for machining. The interface layer was modeled by using cohesive interaction allowing interfacial-damage propagation between the two joint phases as a fracture mechanics phenomenon with a very small thickness (probably 5 μm). Note that the use of a small interface thickness aims to minimize its influence on some other machining responses such as Ti/CFRP chip separation modes, force generation, *etc*. The surface-based traction-separation law with linear softening was adopted to produce the mechanical responses of the cohesive interaction. The failure initiation law required to motivate damage among the interface layer is based on the quadratic stress criterion as illustrated in the following equation.

(10) $${\left(\frac{\sigma_{33}}{t_{n}^{f}}\right)}^{2}+{\left(\frac{\sigma_{13}}{t_{s}^{f}}\right)}^{2}+{\left(\frac{\sigma_{23}}{t_{t}^{f}}\right)}^{2}=1$$

In which, σ_33_, σ_13_ and σ_23_ represent the normal traction stress, and shear traction stresses in two directions, respectively; $t_{n}^{f}$, $t_{s}^{f}$ and $t_{t}^{f}$ denote the peak normal failure strength and peak shear failure strengths in two directions, respectively.

Once the damage onset was satisfied, the Benzeggagh-Kenane (BK) damage criteria [30] and potential law [31] were utilized to simulate damage evolution dominating the cohesive interaction, as presented in Equations (11) and (12). BK criteria are based on the energy dissipated due to failure considering traction-separation responses characterized in terms of released rate energies in the normal and two shear directions (*G_n_*, *G_s_* and *G_t_*).

(11) $$G_{n}^{C}+\left(G_{s}^{C}-G_{n}^{C}\right){\left(\frac{G_{s}+G_{t}}{G_{n}+G_{s}+G_{t}}\right)}^{\eta }=G^{C}$$

(12) $${\left(\frac{G_{n}}{G_{n}^{C}}\right)}^{\beta }+{\left(\frac{G_{s}}{G_{s}^{C}}\right)}^{\beta }+{\left(\frac{G_{t}}{G_{t}^{C}}\right)}^{\beta }=1$$

In which, *G_n_*, *G_s_* and *G_t_* are the released rate energies in the normal and two shear directions respectively; $G_{n}^{C}$, $G_{\text{s}}^{\text{C}}$ and $G_{t}^{C}$ are the critical values of released rate energies, η and β are the parameters of the laws.

In addition, the traction-separation law controlling the responses of the interface zone is specified by means of the stiffness in the normal and in the two shear directions (*K_nn_*, *K_ss_* and *K_tt_*), the interface resistance in each direction ($t_{n}^{f}$, $t_{s}^{f}$ and $t_{t}^{f}$) and the damage evolution through the critical released rate energy ($G_{n}^{C}$, $G_{s}^{C}$ and $G_{t}^{C}$). The input parameters for the interface zone were adopted rigorously based on the comprehensive selection of relevant research works [32,33], as summarized in Table 5.

**Table 5:** Material properties of the interface zone.

Parameter	υ	*K_nn_*	*K_ss_* = *K_tt_*	$t_{n}^{f}$	$t_{s}^{f}$ = $t_{t}^{f}$	$G_{\text{n}}^{\text{C}}$	$G_{\text{s}}^{\text{C}}$ = $G_{\text{t}}^{\text{C}}$
**Value**	0.33	2.0 GPa	1.5 GPa	60 MPa	80 MPa	0.78 N/mm	1.36 N/mm

## 3. Experimental Validation of the OC Model

Due to the significant lack of experimental studies concerning orthogonal cutting of hybrid CFRP/Ti composite in the open literature, the stack model was validated separately in terms of each constituent with experimental results from the literature. Moreover, since the CFRP/Ti interface was considered as a quick transition zone and a very small thickness was defined, its influence on some other machining-induced responses (e.g., Ti/CFRP chip formation mode, force generation, strain/stress) could be ignored. Besides, the input parameters for interface zone were also selected carefully from the literature, in which it had already been validated with experimental results and indicated good suitability for multi-material modeling. Therefore, the validation work was performed solely focused on Ti phase verification and CFRP phase verification by referring to the open literature. Each model was improved and refined carefully until it was capable to replicate consistent results with the experimental observations. For validation purposes, all the numerical simulations were run under the same cutting conditions as used in the literature.

**Figure 3:**
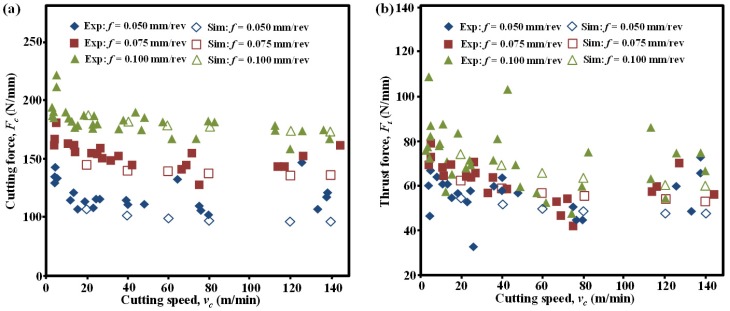
Comparison of the simulated (Sim.) and experimental (Exp.) force generation in Ti-phase cutting modeling [34,35] for different cutting speeds at feeds of 0.05, 0.075 and 0.100 mm/rev: (**a**) cutting force; (**b**) thrust force.

**Figure 4:**
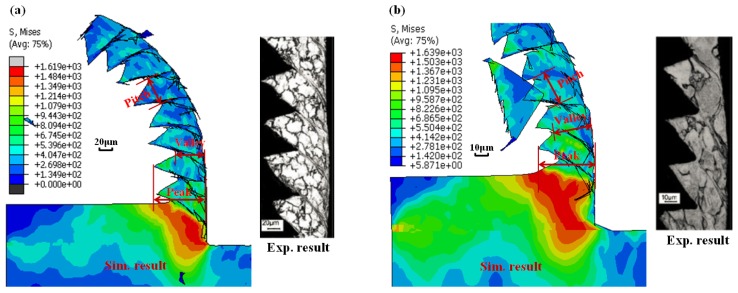
Comparison of the simulated (Sim.) and experimental (Exp.) chip morphologies in Ti-phase cutting modeling [36]: (**a**) test condition 1 (*v_c_* = 1200 m/min and *f* = 70 μm/rev); (**b**) test condition 2 (*v_c_* = 4800 m/min and *f* = 35 μm/rev) (Note: the symbol “S” in the figure represents the von Mises stress and the unit is MPa).

The Ti-phase model was validated rigorously by means of force generation and chip morphology with experimental data from the literature [34,35,36], which were the commonly-used metrics for validations of metal cutting modeling. Figure 3 and Table 6 show the comparison between simulated and experimentally measured force magnitudes [34,35], and the calculated average errors among them, respectively. Note that the force magnitudes (in N/mm) were normalized as the ratio between the average level of force generation and the workpiece thickness. It was apparent that the simulated force generation yielded strong agreement with the experimental measurements for various cutting speeds and feed rates as depicted in Figure 3. Globally, the average errors between the simulated and experimental results were controlled below 10% as summarized in Table 6. Furthermore, comparisons between simulated and experimental chip morphologies were also performed as shown in Figure 4. The serrated chip morphology was validated by three parameters, *i.e*., valley, peak, and pitch, as compared with the experimental results from the literature [36]. As shown in Table 7, the dimensions of the simulated chip morphologies matched well with the experimental ones. The above validations confirmed the credibility of the developed Ti constituent for Ti-phase modeling.

**Table 6:** Average error between the simulated and experimental force generations in Figure 3.

Test Condition	Average Error for Cutting Force (%)	Average Error For Thrust Force (%)
*f* = 0.050 mm/rev	−9.31	−8.32
*f* = 0.075 mm/rev	−6.86	−2.15
*f* = 0.100 mm/rev	+1.73	+1.36

**Table 7:** Comparison between simulated (Sim.) and experimental (Exp.) chip geometries in Figure 4.

Sim./Exp.	Cutting Condition	Chip Morphology (μm)		
		Average Valley	Average Peak	Average Pitch
Simulation	Test condition 1	47.2	95.6	52.3
Experiment		50.3	105.7	68.2
Error		6.16%	9.56%	23.31%
Simulation	Test condition 2	25.4	41.7	27.2
Experiment		23.6	45.6	36.6
Error		−7.63%	8.56%	25.68%

**Figure 5:**
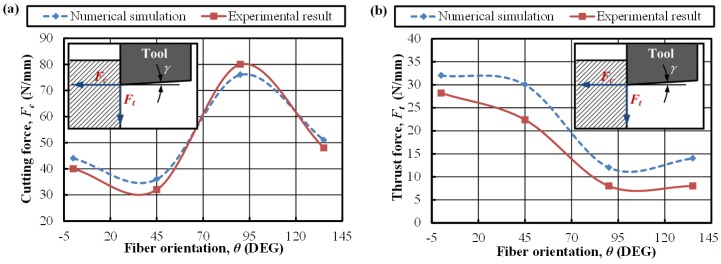
Comparison of the simulated and experimental force generations in CFRP-phase cutting modeling [28]: (**a**) cutting force; (**b**) thrust force (cutting condition: *v_c_* = 6 m/min, *f* = 0.2 mm, α = 0°).

Moreover, the CFRP-phase model was validated through the simplest manner of force generation (cutting force and thrust force) comparison. Figure 5 presents the comparative results of predicted and experimentally measured forces *versus* fiber orientation (θ). It was noticeable that the predicted force magnitudes of both cutting force and thrust force yielded a strong correlation and a consistent variation trend with the experimental results gained by Iliescu *et al*. [28], which confirmed sufficient credibility of the proposed CFRP-phase model.

## 4. Numerical Results and Discussion

In this Section, numerical analyses concerning hybrid CFRP/Ti cutting are presented with the aim of better machining comprehension. Since Ti phase cutting and CFRP phase cutting require different optimal cutting parameters (*v_c_* and *f*), the cutting conditions used in the FE simulations were selected based on the compromise selection of the optimal parametric range for both the two phases of machining. Special focus was made on the analyses of the cutting process, interface damage formation, and parametric effects on fiber/matrix damage extent.

### 4.1. Cutting Process Investigation

**Figure 6:**
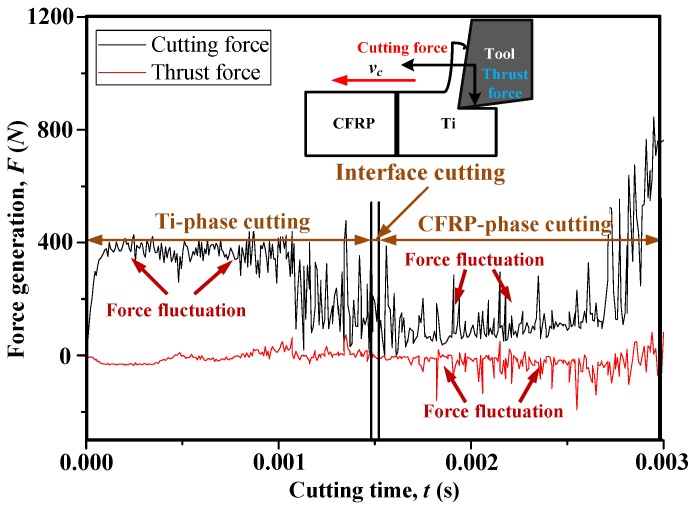
Evolution of the force generation *versus* cutting time when cutting hybrid CFRP/Ti composite (*v_c_* = 40 m/min, *f* = 0.2 mm/rev and θ = 0°).

**Figure 7:**
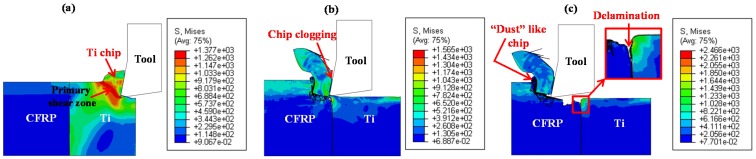
Chip-morphology evolution during CFRP/Ti cutting: (**a**) Ti-phase cutting; (**b**) interface cutting; (**c**) CFRP-phase cutting (*v_c_* = 40 m/min, *f* = 0.2 mm/rev and θ = 0°) (Note: the symbol “S” in the figure represents the von Mises stress and the unit is MPa).

The cutting process of hybrid CFRP/Ti exhibits quite differently from the single-composite and single-metal cutting cases due to the multi-tool-work interaction domains. The disparate natures of each constituent make the chip separation modes more interrelated and coupled governing the bi-material interface consumption (BIC) [37,38]. The interface cutting commonly experienced changeable chip-separation modes and severe transitions of thermo/mechanical responses (e.g., force generation, cutting temperature, strain/stress flow). To reveal the key phenomena controlling CFRP/Ti cutting, the evolution of force generation *versus* cutting time and also the chip formation progression under fixed cutting conditions of *v_c_* = 40 m/min, *f* = 0.2 mm/rev and θ = 0° are presented in Figure 6 and Figure 7, respectively. The force generations in CFRP/Ti cutting were split into two components, *i.e*., the cutting-force component (*F_c_*) and the thrust-force component (*F_t_*), which signify the tribological interactions between tool rake and chip surfaces, together with tool flank and machined surfaces, respectively. It was noticeable that typically three cutting stages referring to the Ti-phase cutting, interface cutting, and CFRP-phase cutting could be seen from the force signal variation and the chip-morphology evolution.

As depicted in Figure 6 and Figure 7, when the tool edges initially cut into the Ti phase, material separation occurred through the elastic-plastic deformation mode that controlled the tool-Ti interaction area. The shearing actions arising from the thermo-mechanical coupling effects produced “continuous” chip morphology that flowed on the tool rake face. It should be noted that the Ti-chip morphology exhibited strong sensitivity to the input parameters (cutting parameters and tool geometries) during machining. Since very low cutting speed and positive tool rake angle were used in the current simulation case, the resected chip shape exhibited a more “continuous” rather than a “serrated” appearance. Under the fixed cutting conditions, the machining operation gradually achieved a steady state for which the forces generation approximately attained a stable variation condition. Besides, despite reaching a steady cutting process, high-frequency force fluctuation was also pronounced in Ti-phase cutting as described in Figure 6. The force-generation usually signifies the mechanical-energy consumption of tool-work interactions in cutting and presents a close relation with the inherent properties of the studied workpiece. Since the Ti alloy exhibited ductile behavior and low thermal conductivity, the chip separation typically involved serious thermo-plastic instability and shear localization in the primary cutting zone. Such a phenomenon would promote the quick occurrence of crack initiation and progression dominating the active cutting zones and result in the instability of the tool-work interaction controlling the chip removal process, as reflected in the cyclic force fluctuations. In addition, when the cutting time (*t*) approximately exceeded 1.0 ms, the cutting-force component was observed to suffer a gradual reduction. The physical phenomenon could be explained by the decreased uncut Ti-chip thickness contributing to the reduction of force resistance when the tool tended to finalize the Ti-phase cutting. With tool advancement, especially when the cutting edges cut into the interface region, the previously-resected Ti chip adhered on the tool rake face and replaced the tool edges for further chip separation. Due to the transition from tool-Ti interaction to tool-CFRP interaction, the cutting force magnitudes underwent quick drop throughout the interface cutting. In such circumstances, the chip separation mode shifted gradually from plastic-defamation into brittle-fracture, which inevitably resulted in the serious transfer of mechanical/physical loads exerted on the tool-work system. The harsh cutting conditions dominating the CFRP/Ti interface cutting was the key contributor promoting the severe damage formation in the bi-material interface.

With further cutting progression, the tool tip cut across the interface region completely and induced a large extent of delamination damage focused on the interface area as shown in the magnified view of Figure 7c. When the tool penetrated into the CFRP phase, material removal took place through successive ruptures aided by the diverse nature and uneven load sharing among the fiber/matrix systems. Since brittle fracture operated as the predominant cutting mode, the resected chip morphology was produced in the form of “discontinuous” shape (especially “dust” like appearance), as illustrated in Figure 7c. Concerning force generation, both the cutting force and thrust force signals were predicted to undergo severe fluctuation and high-frequency variation. However, the mechanisms governing the physical phenomena were disparate. For the cutting force variation, it was induced due to the crack initiation and evolution governing the chip separation process. In contrast, the thrust force fluctuation was mainly attributed to the intense bouncing-back effects on the tool flank surface arising from released carbon fibers in the machined surface [39].

Moreover, when the cutting tool approached the finish of the cutting process, a significant increase of cutting force generation became pronounced as portrayed in Figure 6. This abnormal phenomenon could be explained as follows. When the tool cuts into the CFRP phase, the Ti chip adhered on the tool rake face and replaced the cutting edges for further chip separation. In addition, the produced “dust” like composite chips also caused serious clogging on the head of the Ti chip. Due to these phenomena, the accumulated chip volume inevitably led to a dramatic rise of the cutting resistance, and hence the higher cutting force generation.

### 4.2. Inspection of Interface Damage Formation

**Figure 8:**
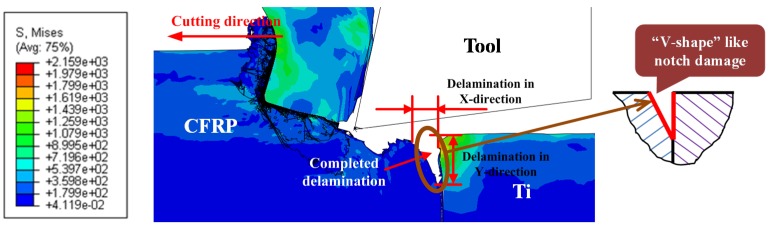
FE observation of interface damage when cutting hybrid CFRP/Ti composite (*v_c_* = 40 m/min, *f* = 0.2 mm/rev and θ = 0°) (Note: the symbol “S” in the figure represents the von Mises stress and the unit is MPa).

**Figure 9:**
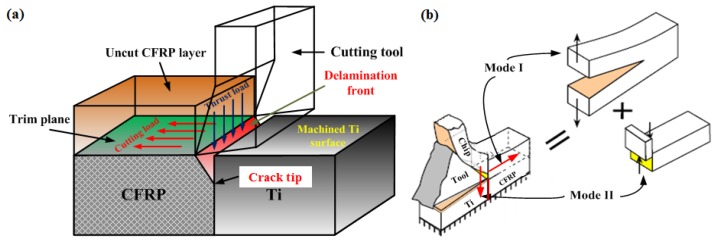
Scheme of (**a**) crack path in interface region and (**b**) fracture modes I and II in cutting.

In CFRP/Ti cutting, the bi-material interface consumption (BIC) signifies the tremendous mechanical/thermal energy transfer arising from disparate phase cutting, the interrelated chip separation modes, and mixed machining responses controlling the hybrid cutting process. BIC plays a significant role in affecting the final surface quality and subsurface damage formation. The interface cutting can be identified as the weakest region vulnerable to serious damage formation. Figure 8 presents the FE observation of interface damage morphology with OC of CFRP/Ti composite under the cutting conditions of *v_c_* = 40 m/min, *f* = 0.2 mm/rev and θ = 0°. It was noticeable that the key characteristic of the interface damage was a “V-shape” like notch damage focused on the CFRP-Ti contact boundary. A large extent of delamination damage became pronounced in both tool-cutting direction (*X*-direction) and through-thickness direction (*Y*-direction) as depicted in Figure 8. However, the key manifestation was the severe tearing in the CFRP-phase boundary deviating far from the Ti-phase boundary. The physical phenomena demonstrated that the crack trajectory would experience a quick damage evolution concerning the tool cutting direction during the machining process. The key mechanisms controlling the crack trajectory and evolution in the bi-material interface were strongly influenced by two important factors. The first was the relative fracture toughness arising from the stacked constituents of the bi-material system. It is believed that the crack path prefers to take place nearer the more brittle constituent characterized by lower fracture toughness since it needs less energy to open and propagate the crack damage. In such a case, the preferred crack path occurring in the CFRP/Ti interface should exhibit more closely to the CFRP-phase boundary due to its relatively lower fracture toughness as compared to its counterpart. Another factor was the specific fracture mode encountered along the bi-material interface. Figure 9 shows the schematization of the crack path and fracture modes I and II in cutting. It could be seen that when the tool edges completely passed through the Ti-phase boundary, the load path exerting on the bi-material system was uniquely applied on the uncut CFRP and interface layers while the machined Ti-phase surface was deprived of load occupation. As such, the cutting load parallel to the tool cutting direction together with the thrust load perpendicular to the cutting direction would produce mixed damage modes (fracture modes I and II) on the interface region. Consequently, it resulted in a sole path evolution of crack trajectory approaching the cutting direction. This phenomenon inevitably gave rise to the so-called “delamination” damage.

**Figure 10:**
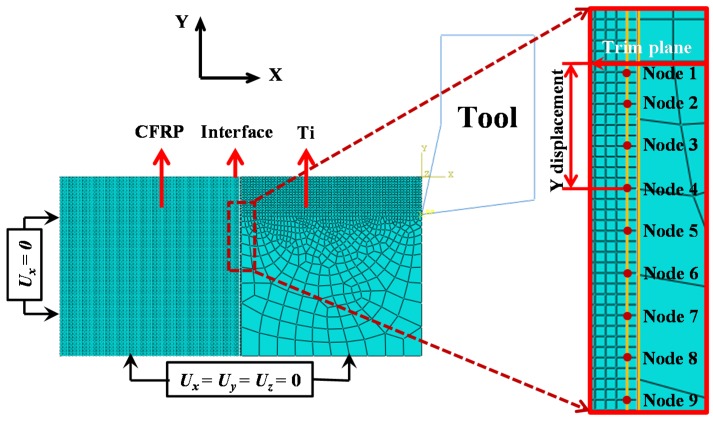
Scheme of selected interface nodes in the CFRP/Ti model.

To specify the dynamic process of delamination formation and also the affected interface zone (AIZ), nine interface nodes beneath the trimmed plane were selected to characterize their *X*-direction displacement *versus* cutting time (*t*) as shown in Figure 10. Table 8 summarizes the depth beneath the trim plane of the selected interface nodes. The AIZ studied here was devoted to clarifying the actual cutting time that influenced the interface damage formation during the orthogonal cutting process.

**Table 8:** *Y*-displacement from the trim plane of selected nodes in the interface zone.

Number of Node	Depth beneath the Trim Plane (μm)	Number of Node	Depth beneath the Trim Plane (μm)
1	5	2	20
3	40	4	60
5	80	6	100
7	120	8	140
9	160	-	

**Figure 11:**
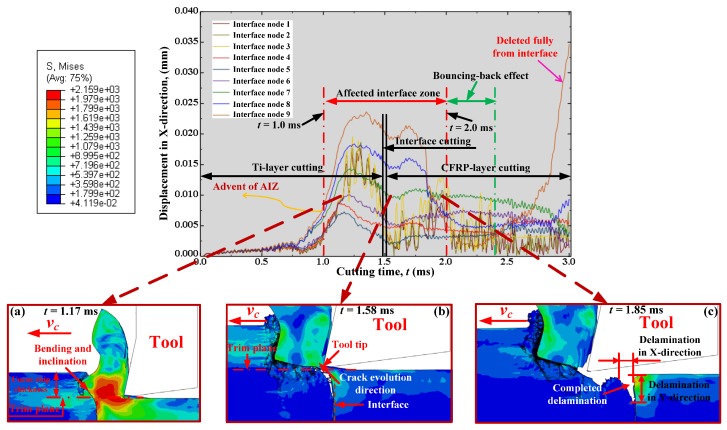
Displacement in *X*-direction of selected interface nodes *versus* cutting time (*t*): (**a**) cutting process at *t* = 1.17 ms; (**b**) cutting process at *t* = 1.58 ms and (**c**) cutting process at *t* = 1.85 ms.

Figure 11 then illustrates the *X* displacement evolution of the selected interface nodes with respect to the cutting time (*t*) under the fixed cutting conditions of *v_c_* = 40 m/min, *f* = 0.2 mm/rev, and θ = 0°. It was apparent that the *X* displacements of the selected interface nodes generally underwent three variation stages, i.e., the initial variation stage, rapid variation stage, and steady variation stage during the total cutting duration (*t* = 3.0 ms). At the beginning of cutting, all the selected interface nodes were predicted to suffer slow-rate displacement variation, which indicated that the chip removal process initially exhibited slight/minor effects on the interface region. However, with the cutting progression, the machining operation gradually exerted significant influences on the output responses of the interface region with evidence that all the selected interface nodes began to experience high-extent displacement fluctuations and quickly enter into the rapid variation stage. The physical phenomena were predicted to take place in the Ti-phase cutting period approximately at a cutting time of 1.0 ms. Such evidence signified the advent of the affected interface zone (AIZ) prior to the interface-cutting period as illustrated in Figure 11. The occurrence of AIZ commonly implied that the CFRP/Ti interface had suffered dramatic influences arising from the Ti-chip removal process. As depicted in Figure 11a, the produced Ti chip caused severe bending and inclination effects on the interface zone and also the uncut CFRP-chip layer. The pronounced displacement variation in AIZ strongly demonstrated the appearance of serious detaching and separation concerning the interface zone. In addition, the interface nodes located near the trim plane typically underwent larger *X* displacement compared to their counterparts far away from the trim plane. The reason might be due to the different levels of influences arising from tool-work interactions. When the cutting time approximately reached 2.0 ms, the *X* displacements of the selected interface nodes gradually achieved the steady variation state, indicating the diminishing trend of the cutting influences on the interface region. For instance, as shown in Figure 11c, both the deamination lengths in *X* and *Y* directions reached their stable values. In such a case, the AIZ could be defined probably at a duration of *t* ∈ [1.0, 2.0 ms]. With regard to the severe displacement fluctuation occurring in the steady variation state, the reason might be induced as being due to the bouncing-back effects from adjacent CFRP elements still affected by the local cutting operation. At the end of the cutting process, all the selected interface nodes approached attainment of constant displacements in the *X* direction except the interface node 1. The dramatic displacement increase of interface node 1 demonstrated that the node had been deleted completely from the interface zone.

**Figure 12:**
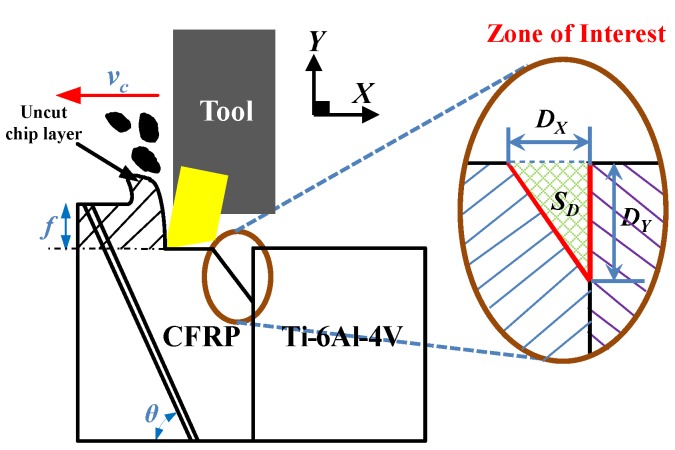
Scheme of delamination damage evaluation in CFRP/Ti cutting.

To quantify the delamination extent occurring in the interface zone, three indicators, *i.e*., *D_X_*, *D_Y_*, and *S_D_* were introduced for evaluation in this study, where *D_X_* signified the delamination length in the tool-cutting direction (*X*-direction), *D_Y_* denoted the delamination length in the through-thickness direction (*Y*-direction) and *S_D_* implied the delamination area of the damage zone as shown schematically in Figure 12. The delamination area (*S_D_*) was defined as illustrated in Equation (13).

(13) $$S_{D}=0.5\times D_{X}\times D_{Y}$$

**Figure 13:**
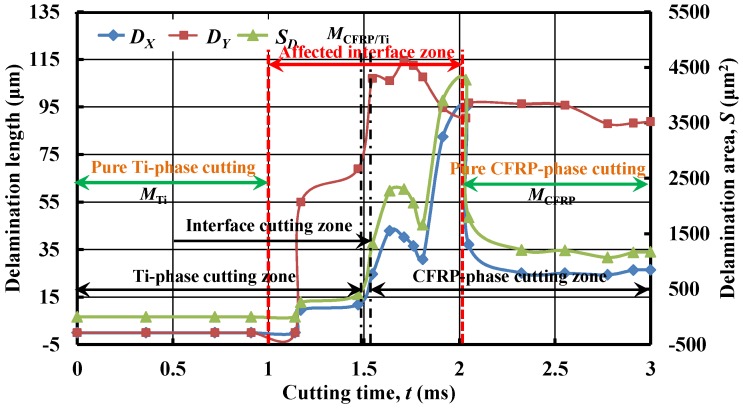
Evolution of delamination damage (*D_X_*, *D_Y_* and *S_D_*) as function of cutting time (*t*).

Figure 13 presents the evolution of interface delamination damage *versus* cutting time (*t*). The results confirmed that all the delamination indicators (*D_X_*, *D_Y_* and *S_D_*) exhibited similar evolution trends as the *X* displacements of the selected interface nodes *versus* cutting time (*t*) in Figure 11. The comparison mutually demonstrated the reliability of the two numerical observations. Moreover, as depicted in Figure 13, from the geometrical consideration, the entire hybrid CFRP/Ti cutting can be divided into three stages referring to (*i*) Ti-phase cutting, (*ii*) interface cutting, and (*iii*) CFRP-phase cutting in terms of their respective cutting lengths. However, such a category actually couldn’t reflect the real machining behavior of the bi-material system due to ignorance of the interactive cutting influences arising from each-phase cutting. In reality, the entire hybrid cutting process could be divided into three basic zones based on the interactive influences of each phase machining.

(*i*) Pure Ti-phase cutting *t* ∈ [0, 1.0 ms] signifies the cutting period concerning the absolute Ti-phase machining with minimum influence affecting the CFRP/Ti interface and the CFRP zone. Such a zone represents the machinability of the single Ti-alloy phase (*M*_Ti_).

(*ii*) AIZ cutting *t* ∈ [1.0, 2.0 ms] denotes the cutting duration concerning the machining variation of the bi-material interface arising from Ti-phase cutting and CFRP-phase cutting. Moreover, the AIZ cutting zone in reality represents the real machinability of the stacked CFRP/Ti material (*M*_CFRP/Ti_).

(*iii*) Pure CFRP-phase cutting *t* ∈ [2.0, 3.0 ms] implies the cutting time concerning the absolute CFRP-phase machining and reflects the machinability of the standard CFRP phase (*M*_CFRP_).

During the pure Ti-phase cutting zone, all *D_X_*, *D_Y_* and *S_D_* nearly remained constant values (probably zero values), indicating that no delamination occurred in the interface zone. With the continuation of the cutting process, especially when *t* reached the AIZ, the three delamination indicators totally suffered a dramatic increase with elevated cutting time. Such a phenomenon signified that the delamination damage was principally formed at the AIZ cutting period. Besides, the subsequent reduction of the delamination indicators in the AIZ might be caused by the bouncing-back effects from adjacent CFRP elements affected by the cutting operation. Moreover, with further increased *t*, especially in the pure CFRP-phase cutting zone, *D_X_*, *D_Y_* and *S_D_* gradually reached their steady values, which meant that the later cutting process (post CFRP-phase cutting) generated less effect on the interface delamination formation.

**Figure 14:**
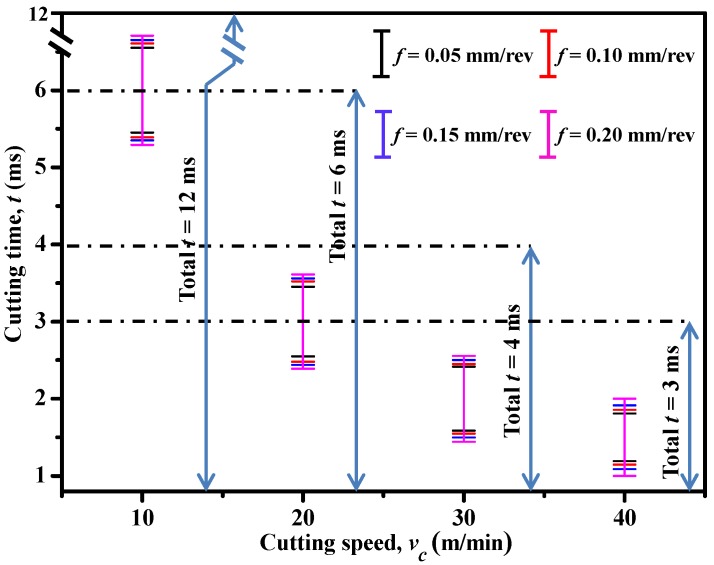
Effects of cutting speed (*v_c_*) and feed rate (*f*) on cutting duration of AIZ (Note: “**Ι**” indicates the cutting duration of AIZ) (θ = 0°).

**Figure 15:**
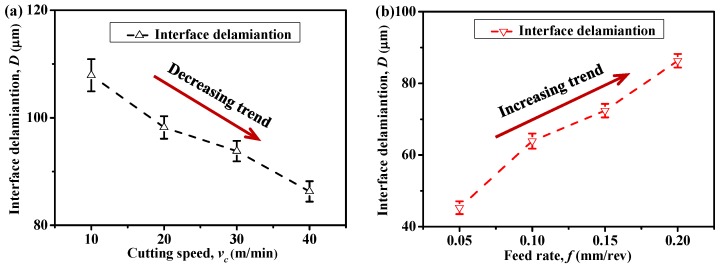
Interface delamination extent (*D*) in function of: (**a**) cutting speed (*v_c_*) (*f* = 0.2 mm/rev and θ = 0°) and (**b**) feed rate (*f*) (*v_c_* = 40 m/min and θ = 0°).

Moreover, to inspect the effects of input variables on the AIZ and also on the interface delamination extent, a parametric study was performed by considering various cutting speeds and feed rates. Figure 14 and Figure 15 present the acquired results. Note that in Figure 14, the symbol “**I**” indicates the cutting duration of AIZ and the interface delamination extent (*D*) was measured based on the *D_Y_* length (delamination length in the through-thickness direction (*Y*-direction)). As can be seen in Figure 14, both *v_c_* and *f* were found to have significant effects on the AIZ cutting time. Specifically, *v_c_* exhibited a negative impact on the AIZ cutting duration while the impact of *f* was positive. A parametric combination of high *v_c_* and low *f* commonly resulted in the lowest AIZ cutting duration. Moreover, as is evident in Figure 15, all the examined parametric variables showed pronounced influences on the interface delamination extent. The impact of *v_c_* was found to be negative, i.e., an increase of *v_c_* resulted in the reduction of *D*, while the impact of *f* was positive. The mechanisms controlling the variation phenomena could be associated with their specific influences on AIZ. This was because when *v_c_* was elevated, typically a reduced cutting time of AIZ was achieved as illustrated in Figure 14. As such, the decreased AIZ inevitably alleviated the damage extent of induced interface delamination. In contrast, an increase of *f* usually led to an increased AIZ (as depicted in Figure 14), and hence gave rise to a higher extent of interface delamination as shown in Figure 15b. Therefore, from the above analyses, for minimizing the extent of interface delamination, a high cutting speed and a low feed rate are strongly preferred.

### 4.3. Subsurface Damage Study

**Figure 16:**
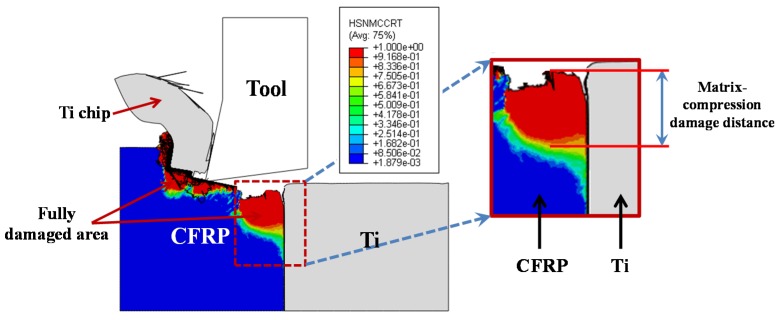
Scheme of damage measurement in the CFRP phase (Note: HSNMCCRT represents the matrix-compression failure mode).

**Figure 17:**
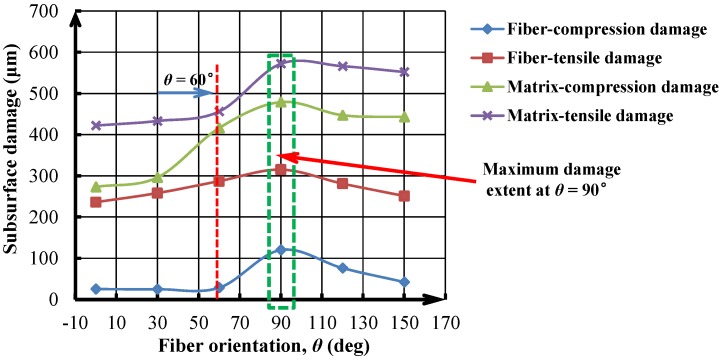
Fiber/matrix damage extent *versus* fiber orientation (θ) (*v_c_* = 40 m/min and *f* = 0.2 mm/rev).

Apart from the severe interface imperfection, the subsurface damage occurring in the composite phase is also a particular concern when machining hybrid CFRP/Ti composite. As discussed above, the composite-phase damage takes place following four types of fiber/matrix failure, *i.e*., fiber compression damage, fiber tensile damage, matrix compression damage, and matrix tensile damage. To inspect the subsurface composite damage resulting from the hybrid cutting operation, a parametric analysis was performed. The fiber/matrix failure was measured as the largest distance from the machined CFRP surface to the deepest fully damaged area, as shown schematically in Figure 16. The fiber/matrix failure extent was predicted and studied *versus* the fiber orientation (θ) under the fixed cutting conditions of *v_c_* = 40 m/min and *f* = 0.2 mm/rev as depicted in Figure 17. It was noticeable that the *θ* exhibited significant influence on the evolution of the various types of fiber/matrix damage during the chip removal process. The subsurface damage extent increased with elevated θ, especially the elevation became pronounced when θ was above 60° as depicted in Figure 17. All the damage types tended to reach their maximum extent at θ = 90°. With θ’s further increase, the subsurface damage appeared to suffer a slight decrease. Such findings of θ’s effect on composite damage agreed well with the observations of Arola *et al*. [17], and Wang and Zhang [39] when cutting CFRP laminates. The mechanisms controlling the mentioned phenomena could be attributed to the change of failure modes from bending and crushing in case of θ = 0° to a fracture by compression and interfacial-shearing mode for positive fiber orientation (0° < θ ≤ 90°) [40], which inevitably led to in-depth fiber/matrix damage. For θ above 90°, chip-separation modes were shifted to be dominated by pressing, inter-laminar shearing, matrix cracking, and fiber/matrix interface debonding. This situation would result in less energy-consumption for chip separation and lower cutting resistance for machining. As a result, less extent of damage formation was promoted.

**Figure 18:**
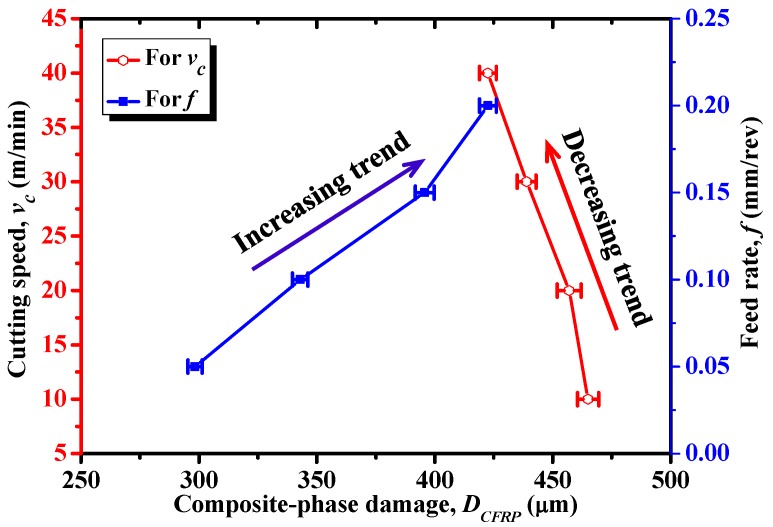
Effects of cutting speed (*v_c_*) and feed rate (*f*) on composite-phase damage extent (*D*_CFRP_) (θ = 0°).

In addition, Figure 18 also shows the parametric effects of *v_c_* and *f* on the cutting-induced composite-phase damage. Note that the composite-phase damage (*D*_CFRP_) was evaluated based on the type of fiber/matrix failure that caused the largest extent of damage (e.g., as shown in Figure 17, matrix-compression damage caused the maximum damage extent). Afterward, *D*_CFRP_ was measured as the largest distance from the machined CFRP surface to the deepest fully damaged area. Each measurement was repeated three times in order to ensure sufficient credibility for the acquired results. As shown in Figure 18, it was noticeable that the *f* had remarkable effects on the composite-phase damage in such a manner that a small increase of *f* resulted in a dramatically elevated *D*_CFRP_. In contrast, an increase of *v_c_* typically led to a direct reduction of *D*_CFRP_. The phenomena implied that the use of high-speed cutting (HSC) might benefit the reduction of composite-phase damage formation when machining hybrid CFRP/Ti composite. Therefore, based on the above analyses, the optimum cutting parameters for composite damage minimization should consist of small fiber orientation, high cutting speed, and low feed rate when machining this bi-material system.

## 5. Conclusions

In this paper, a finite element model was developed to simulate the chip formation process with orthogonal cutting of hybrid CFRP/Ti composite. The proposed OC model attempted to address the fundamental mechanisms dominating hybrid composite machining. The numerical studies provided a better comprehension of various cutting phenomena induced in the hybrid cutting process. Based on the results acquired, key conclusions can be drawn as follows.

(1) The disparate natures of each stacked constituent resulted in the hybrid cutting process exhibiting three distinct cutting stages, *i.e*., Ti-phase cutting, interface cutting, and CFRP-phase cutting. The chip separation modes controlling the bi-material cutting comprise both elastic-plastic deformation and brittle fracture. The changeable chip removal mechanisms typically resulted in extremely harsh conditions dominating the interface cutting. Severe force fluctuation was observed as the main characteristic governing the hybrid CFRP/Ti cutting. Serious chip adhesion on the tool rake face was found to be the key factor contributing to the dramatic increase of cutting force generation in post CFRP-phase machining.

(2) Numerical results highlighted the occurrence of severe delamination damage concerning the CFRP/Ti interface. FE analyses revealed that the initiation and evolution of interface delamination primarily took place at the cutting period of AIZ. The formation of CFRP/Ti interface damage involved a series of detaching and tearing variations governing the AIZ cutting time. The key mechanisms governing the interface damage formation could be attributed to the coupling effects of fracture modes I and II arising from the unique mechanical loads exerted on the bi-material interface region. The key morphological characteristic of the interface delamination was a “V-shape” like notch focused on the CFRP-Ti contact boundary.

(3) Parametric studies confirmed the significant role of the cutting speed and feed rate in affecting the AIZ cutting duration. The input variables (*v_c_* and *f*) indeed exhibited pronounced effects on the interface delamination extent via the manner of their influences on the AIZ. For minimizing severe interface damage formation, a parametric combination of high cutting speed and low feed rate is strongly preferred.

(4) The fiber/matrix damage promoted in hybrid CFRP/Ti cutting was carefully studied *versus* input variables. The numerical results pointed out the crucial role of fiber orientation, feed rate, and cutting speed in affecting induced composite-phase damage. For composite-phase damage minimization, the optimum cutting parameters should comprise small fiber orientation, high cutting speed, and low feed rate when machining hybrid CFRP/Ti composite.

(5) Another key finding revealed in this paper showed that the entire hybrid cutting could be physically divided into three cutting zones from the viewpoint of the interrelated influences arising from each-phase machining: (*i*) pure Ti-phase cutting, (*ii*) AIZ cutting and (*iii*) pure CFRP-phase cutting. Also, among them, the AIZ cutting in reality reflects the real machinability of the stacked CFRP/Ti material (*M*_CFRP/Ti_), which can be considered as the most difficult-to-cut zone due to the occurrence of interrelated cutting influences and the existence of the weakest interface zone. In such a case, to fundamentally improve the machinability of the hybrid CFRP/Ti composite, special focus should be made concerning the in-depth mechanism investigation and cutting-parameter optimization of the AIZ cutting zone. In the future, more systematic studies should be performed to address precisely the aforementioned issues.

## Abbreviations

The following abbreviations are used in this manuscript:

AIZ: Affected interface zone
BIC: Bi-material interface consumption
CFRP: Carbon Fiber Reinforced Polymer
CZ: Cohesive zone
*D*: Interface delamination extent
*D*_CFRP_: Composite-phase damage
*D_X_*: Delamination length in the tool-cutting direction (X direction)
*D_Y_*: Delamination length in the through-thickness direction (Y direction)
EHM: Equivalent homogeneous material
*f*: Feed rate
*F_c_*: Cutting force
FE: Finite element
*F_t_*: Thrust force
JC: Johnson-Cook
M_CFRP_: Machinability of pure CFRP-phase cutting
*M*_CFRP/Ti_: Machinability of stacked CFRP/Ti composite
*M*_Ti_: Machinability of pure Ti-phase cutting
OC: Orthogonal cutting
*S_D_*: Delamination area of the damage zone
α: Tool rake angle
θ: Fiber orientation
γ: Tool clearance angle
*t*: Cutting time
*v_c_*: Cutting speed
